# UHF RFID Prototyping Platform for ISO 29167 Decryption Based on an SDR

**DOI:** 10.3390/s19102220

**Published:** 2019-05-14

**Authors:** Georg Saxl, Manuel Ferdik, Moritz Fischer, Martin Maderboeck, Thomas Ussmueller

**Affiliations:** Microelectronics and Implantable Systems Group, Department of Mechatronics, University of Innsbruck, Innsbruck 6020, Austria; manuel.ferdik@uibk.ac.at (M.F.); moritz.fischer@uibk.ac.at (M.F.); martin.maderboeck@student.uibk.ac.at (M.M.); thomas.ussmueller@uibk.ac.at (T.U.)

**Keywords:** cryptography, data security, radio frequency identification, software defined radio, UHF communication

## Abstract

Ultra high frequency radio frequency identification (UHF RFID) is becoming a key technology in the Internet of Things. It allows the implementation of batteryless and wireless nodes, including sensors and actuators. Due to its possible transmission range of >10 m and potential to carry critical information, security is a highly important topic. For this reason, the International Organization for Standardization has published several crypto suites for UHF RFID within the ISO-29167 standard in 2014. Recently, research has focused on implementing those encryption features on the transponder side. However, currently no crypto enabled UHF RFID readers are available. In order to cope with the rapid development in this field, ‘open’ and flexible readers based on software defined radios are needed. They make it possible to quickly adapt the protocol and to test new functionalities such as encryption. This paper deals with the first implementation of the ISO 29167-19 standardized RAMON decryption on a software defined radio. The programming of this hardware is done in LabVIEW which allows for controlling the built-in transceiver modules. However, first measurements show that the decryption takes 51 s. This is because LabVIEW is not suitable for handling very large numbers like they are utilized in cryptography. Because such a long processing time is not feasible in experiments nor in a real-life scenarios, this method is not suitable for a prototyping platform. Thus, a different approach is chosen to optimize the decryption processing time. LabVIEW still provides the framework for handling the protocol and controlling the transceivers, but the decryption is performed in a Java application. In that way, the entire decryption process takes only about 2.2 ms, which is 23,318 times faster than the implementation in LabVIEW. Thus, this new approach meets the necessary timing requirements and is suitable for realistic application scenarios. The shown method allows development and testing of new functionalities in UHF RFID systems but may also be employed in any application that require long processing times in LabVIEW. Furthermore, the implementation of decryption features is the first necessary step towards a fully compliant, crypto enabled interrogator for UHF RFID, featuring a high adaptability.

## 1. Introduction

The Internet of Things (IoT) is a vision of the future that is currently attracting a lot of attention. It describes a network of billions of devices, sensors and gadgets interconnected via embedded, digital communication interfaces. This allows quick and decentralized information processing, execution, sharing and broadcasting. Typically, wireless connections between nodes are preferred in IoT, due to reduced installation efforts, higher flexibility and often reduced costs [1]. However, wireless systems have the disadvantage that the power supply cannot be ensured via a cable. Therefore, either batteries are needed, which are to be avoided from an environmental and maintenance point of view, or the energy is generated locally on the wireless device itself. Thus, batteryless and wireless systems are a very interesting option. One of the best known representatives is “Ultra High Frequency Radio Frequency Identification (UHF RFID)”.

The original idea of this technology is contactless identification of goods or persons, which is demonstrated in Ref. [2]. However, recent research also deals with sensors based on UHF RFID, which can be used to determine physical parameters of the environment. This is an extremely exciting development with regard to the advancing digitization in the course of Industry 4.0 as well as in home automation and many other areas [3]. Due to battery-free wireless operation, the sensors are maintenance-free and can be used almost anywhere, even in inaccessible places such as inside components. Another recently discussed application of UHF RFID is traffic management and intelligent parking, where cars can be labeled with passive UHF RFID tags to allow, for example, direct billing of parking costs or better planning of traffic flows [4,5].

Due to the advantages of UHF RFID, the technology has become widespread in recent years. This is accompanied by a growing need for security [6,7]. Especially due to the fact that UHF RFID allows ranges of more than 10 meters, the security of the transmitted data is even more crucial than with near field communication systems. Possible implementations of encryption for RFID have already been shown several times [8,9,10]. The problem with UHF RFID is the low energy available at the passive transponder [11]. Therefore, special attention must be paid to low complexity of the encryption method to reduce hardware and energy consumption. Suitable approaches for tag side encryption such as the use of Elliptic Curve Cryptography have already been presented in Refs. [12,13]. In Ref. [14], a tag prototype is presented, which demonstrates RAMON encryption on a Field Programmable Gate Array (FPGA). However, the authors postpone detailed measurements and a silicon implementation until compatible readers are available. Until 2014, there was no standardized encryption procedure for UHF RFID. With the ISO 29167 standard, this has changed. It provides nine different Crypto Suites including AES-128, PRESENT-80 and RAMON.

To cope with the rapid development of UHF RFID both in cryptography and other areas such as passive sensors and actuators [15], “open” (easy adaptable) readers in the form of prototyping platforms are required. To keep up with rapid development, it is important that both hardware and software changes can be made quick and easily. Software defined radios (SDR) are one way to meet this requirement as shown in Refs. [16,17].

With this paper, we present the first SDR based prototyping platform with a time-optimized implementation of the ISO 29167-19 standardized RAMON decryption. An NI PXIe-1075 chassis is chosen as the hardware platform, which is equipped with a transceiver module PXIe-5644R. The implementation of a basic reader on this platform has already been demonstrated [16]. The RAMON procedure is well suited for UHF RFID because the encryption on the transponder side is resource-saving and the main processing work occurs on the reader side. However, this leads to the fact that the reader takes a very long time to decrypt if not properly implemented. Therefore, the time efficiency is taken into account in the following procedure.

## 2. RAMON Crypto System

The RAMON crypto system is a mixture of the “RAbin crypto system” [18] and the “MONtgomery multiplication” [19]. The Rabin crypto system is an asymmetric encryption technique whose security is based on the factorization problem. In combination with the Montgomery multiplication, it is used to achieve a low power consumption for encryption, but with a higher effort needed for decryption. Therefore, this method is particularly suitable for UHF RFID systems, since little energy and thus little computing power are available on the passive node (transponder), but, in proportion, a lot more energy and computing power is available on the reader side. This paper focuses on the reader side decryption and implementation on an SDR. For a better understanding, the encryption [20] is described briefly in the following section.

### 2.1. Encryption

**Figure 1:**
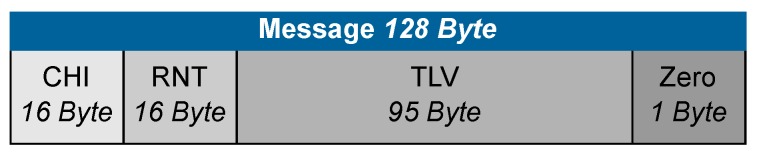
Format of the plain message *x* which will be processed by the RAMON crypto system.

**Figure 2:**
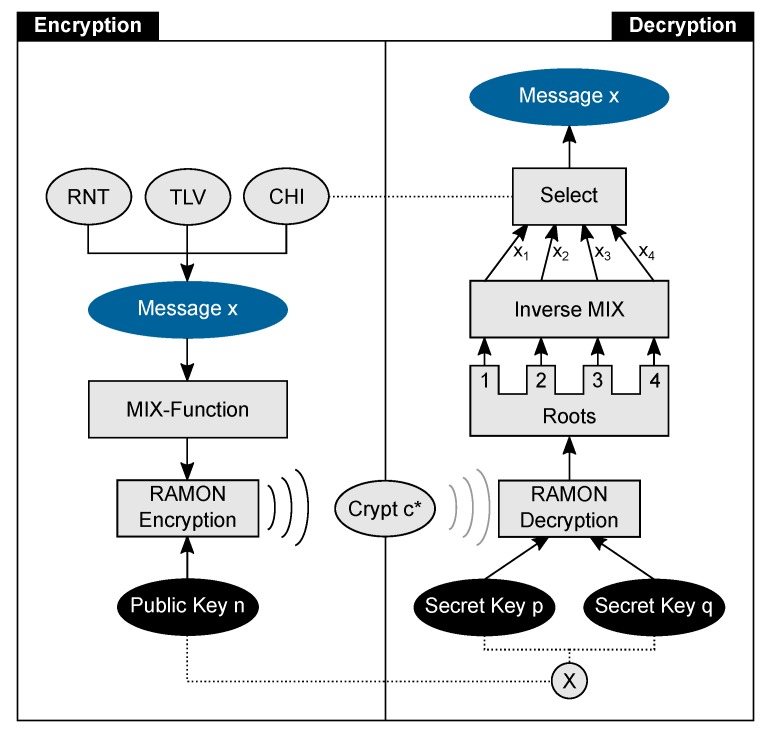
Schematic representation of the RAMON crypto system process. The plain message *x* is mixed and then RAMON encrypted. This requires the public key *n*, which has already been pre-transmitted from the reader to the tag. The Crypt $c^{*}$ is transmitted to the reader via the air interface and decrypted with the help of the secret keys *p* and *q*. There are four possible solutions to choose from. Since the reader already knows CHI, it can be used to identify the actual plain message and to select the correct *x*.

The plain message *x* shown in Figure 1 has a total length of 128 bytes and consists of 16 bytes CHI (challenge interrogator), 16 bytes RNT (random number tag), 95 bytes TLV (tag length value) and 1 byte zero padding. This message will be processed as shown in the flow chart in Figure 2. First, the message is mixed with a specific pattern to further enhance security.

To encrypt the given message, the public key *n*, which is the product of the secret keys *p* and *q*, has to be pre-transmitted and already known at the transponder. The Rabin crypto system calculates the crypt *c*, which is the result of encryption performed on the mixed message *x* with the public key *n*:

$$c=x^{2}\;\mathrm{mod}\;n.$$

In order to avoid the power-intensive modulo operation the Montgomery multiplication is implemented within the RAMON crypto system. Its purpose is to calculate $c^{*}$, which is defined as

(1) $$c^{*}=x^{2}\;R^{-1}\;\mathrm{mod}\;n.$$

The Residuum *R* is defined as well chosen power of two (in case of RAMON R=1088). Equation (1) is calculated by the efficient Montgomery multiplication algorithm, which contains only simple arithmetic operations and is therefore very power-efficient.

### 2.2. Decryption

The simplicity of encryption via the efficient Montgomery multiplication has to be compensated by a more complex decryption. Basically, the reader has to calculate the square roots of the problem to receive the decrypted message *x*. Thus, it is necessary to transform the crypt $c^{*}$ back to *c* by

(2) $$\begin{array}{ccc} c & = & c^{*}\;R\;\mathrm{mod}\;n\\ & = & x^{2}\;R^{-1}\;R\;\mathrm{mod}\;n\\ & = & x^{2}\;\mathrm{mod}\;n. \end{array}$$

Next, the inverse elements of the secret keys $p_{i}$ an $q_{i}$ have to be determined by the “Extended Euclidean Algorithm” [21] so the following equation is valid:

(3) $$p_{i}\;\cdot \;p+q_{i}\;\cdot \;q=1.$$

To calculate the square root of *c* an intermediate step is needed, which first calculates the square root of cmodp and cmodq resulting in

(4) $$\begin{array}{c} a_{p}=c^{\frac{p+1}{4}}\;\mathrm{mod}\;p, \end{array}$$

(5) $$\begin{array}{c} a_{q}=c^{\frac{q+1}{4}}\;\mathrm{mod}\;q. \end{array}$$

With $a_{p}$ and $a_{q}$, it is possible to determine the four square roots modulo *n* via the “Chinese Remainder Theorem” (CRT) [22]. One of those solutions is the correct decrypted message *x*:

(6) $$\begin{array}{c} x_{1}=\left(p_{i}\;\cdot \;p\;\cdot \;a_{q}+q_{i}\;\cdot \;q\;\cdot \;a_{p}\right)\;\mathrm{mod}\;n, \end{array}$$

(7) $$\begin{array}{c} x_{2}=n-x_{1}, \end{array}$$

(8) $$\begin{array}{c} x_{3}=\left(p_{i}\;\cdot \;p\;\cdot \;a_{q}-q_{i}\;\cdot \;q\;\cdot \;a_{p}\right)\;\mathrm{mod}\;n, \end{array}$$

(9) $$\begin{array}{c} x_{4}=n-x_{3}. \end{array}$$

After inverse mixing all four solutions, the correct plain message is determined by the CHI which is part of the message and is known by the reader.

## 3. Basic Implementation in LabVIEW

As a hardware platform the software defined radio from NI was chosen. It consists of a PXIe-1075 chassis, a PXIe-8135 controller and a PXIe-5644R transceiver module. The implementation of a UHF RFID reader using this platform and the graphical programming in LabVIEW has already been shown in [16]. In order to integrate the decryption seamlessly into the existing reader protocol, LabVIEW is also utilized for the implementation of the RAMON procedure. Since LabVIEW is only able to handle numbers with a maximum of 64 bit, the standard calculations (add, multiply, divide, …) are not suitable for cryptography where numbers of 1024 bits and more are used. Due to this fact and the lack of a data type that can handle numbers of this size in LabVIEW, it is necessary to develop an own library which contains all needed operations:

- Big Binary operations: Basic arithmetic operations,
- RAMON crypto system: Equations for decryption,
- Array conversion: Conversion of test vectors and results.

**Figure 3:**
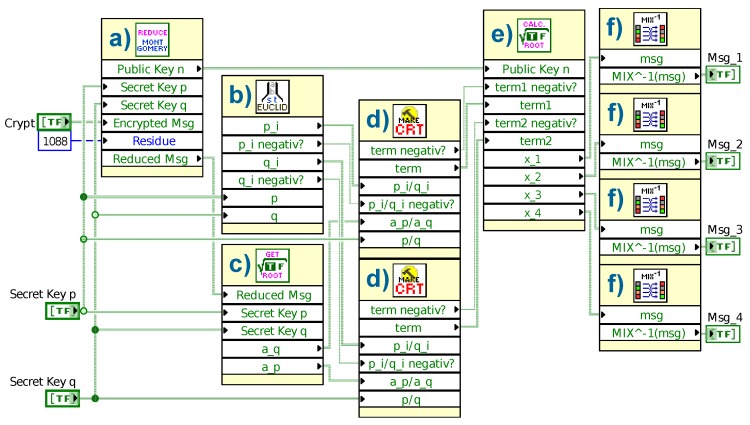
Decryption process implemented in LabVIEW: The crypt $c^{*}$ and both secret keys *p* and *q* are inputs. The four possible solutions are computed by the LabVIEW-SubVI Blocks: (**a**) Reduce Montgomery; (**b**) Extended Euclidean Algorithm; (**c**) Get-Root; (**d**) make CRT-Term; (**e**) Calculate Root; (**f**) Inverse MIX-Function.

With these basic operations, it is possible to rebuild the equations given in Section 2.2 and thus construct a LabVIEW program for the RAMON decryption as shown in Figure 3. The correlations of the functions (a) to (f) from Figure 3 and the Equations (3)–(9) are shown in Table 1.

### 3.1. Runtime Optimization of Arithmetic-Algorithms

After basic tests with test vectors taken from the ISO 29167-19 standard [20], very long computation times have been measured. To achieve an efficient decryption, the operation time has to be cut down. Therefore, faster algorithms have to be used for basic binary arithmetic which are described in the following.

#### 3.1.1. Ripple Carry Adder

The most used arithmetic block by all hierarchical higher SubVIs (RAMON-Blocks) is by far the “ADD” Algorithm. To enhance the performance of all other blocks, the add algorithm has to be optimized. This is achieved with the use of a “Ripple Carry Adder” [23]. It is based on an XOR operation on both addends a[] and b[] as well as on a third array c[] which contains carry bits. The result s[] is defined as

s[i]=a[i]⊕b[i]⊕c[i].

The carry array c[] is initialized with c[0]=0 and all further entry’s are calculated with

$$c\left[i+1\right]=\left(c[i]\wedge (a[i]⊕b[i])\right)\vee \left(a\left[i\right]\wedge b\left[i\right]\right).$$

#### 3.1.2. Multiply

**Figure 4:**
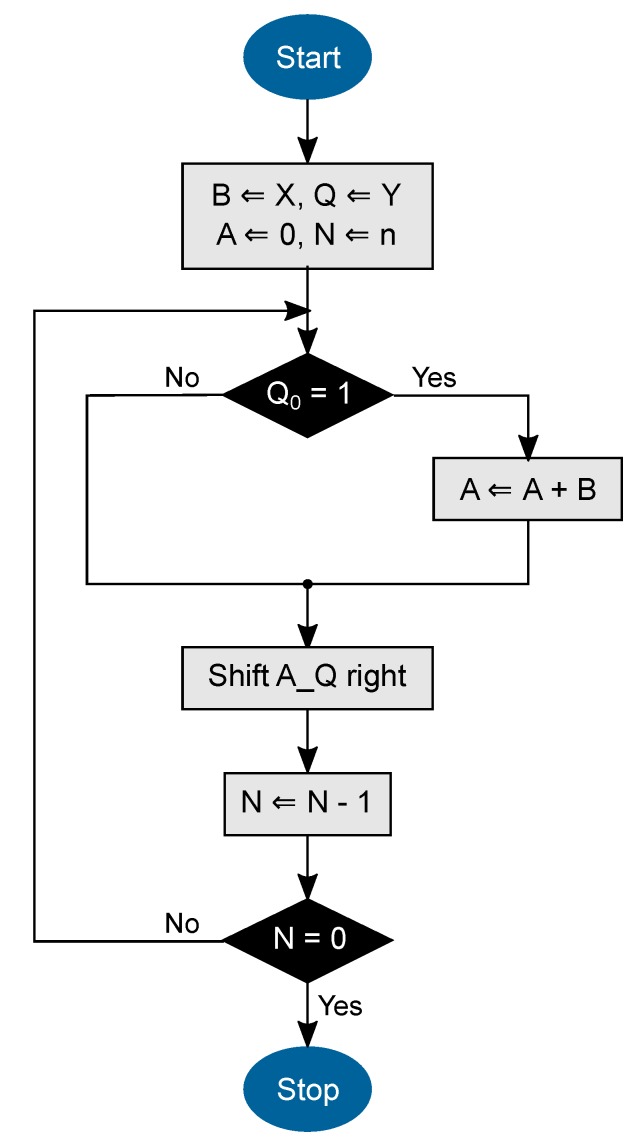
“Shift-and-Add” multiplication algorithm based on Ref. [24]. The aim is to multiply the *n*-bit long numbers *X* and *Y*. The product register *A* is initialized with 0 and the counter *N* with *n*.

The multiplication of two large numbers is implemented by using the “Shift-and-Add” procedure mentioned in Ref. [24]. The flow diagram of the algorithm is shown in Figure 4. It calculates the product of the two *n*-bit long numbers *X* and *Y*. Therefore, the multiplicand *X* is added to itself *Y* times. As a result, the algorithm delivers the 2n-bit long product register *A*.

#### 3.1.3. Quotient and Remainder—Modulo

Another important arithmetic operation for cryptography is the modulo operation. The LabVIEW implementation is based on the “Quotient and Remainder” theorem shown in Algorithm 1. The pseudo-code is similar to the “Multiple-precision division” algorithm from Ref. [25]. To calculate quotient *q* and remainder *r* of a division x/n, it is possible to subtract x−n exactly *q*-times until

r=x−n·q<n.

The algorithm shown in Algorithm 1 basically manipulates the divisor *n* by left-shifting (multiplication with power of two) until the most significant bit (MSB) of the boolean array is at the same position as the MSB of *x*. Therefore, it is possible to reduce *x* by power-of-two times at once, which results in a faster solution for the problem than step-wise subtraction. How often *x* has been shifted is noted and transferred to *q*.
**Algorithm 1:** Pseudo Code of the ‘Quotient and Remainder’ algorithm to calculate the modulo of two numbers.
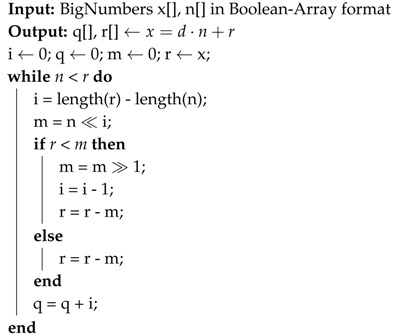


#### 3.1.4. Modular Exponentiation—ModPower

The “Modular Exponentiation” or “Mod-Power” calculation is primarily used by Block (c) “Get Root” from Figure 3. It computes

$$r=x^{e}\;\mathrm{mod}\;n.$$

When $x^{e}$ becomes a huge number, it is necessary to split up the calculation. By increasing the *x* exponential and dividing it by *n*, an overly large number can be avoided as an intermediate result. To calculate the result of *x* partially exponential, an algorithm called “Exponentiation by squaring” [26] is used. It considers *e* as binary number and for each 0 (starting from MSB) *x* gets squared (‘*S*’) and for every 1 in *e* *x* gets squared and multiplied (‘SM’) by *x*. Equation (10) shows an example for $x^{11_{10}}=x^{1011_{2}}$. Therefore, the exponent $e=11_{10}=1011_{2}$ causes 1011→SMSSMSM. The first SM only means $1^{2}\;\cdot \;x$ and thus can be canceled and started with *x*:

$$x\overset{\exp(2)}{\underset{S}{\to }}x^{2}\overset{\exp(2)}{\underset{S}{\to }}x^{4}\overset{\;\cdot \;x}{\underset{M}{\to }}x^{5}\overset{\exp(2)}{\underset{S}{\to }}x^{10}\overset{\;\cdot \;x}{\underset{M}{\to }}x^{11},$$

(10) $$x^{11_{10}}=x^{1011_{2}}={\left({\left(x^{2}\right)}^{2}\;\cdot \;x\right)}^{2}\;\cdot \;x.$$

After each intermediate step (‘*S*’ and ‘SM’), it will be divided by *n* and continued with the remainder to keep the result a small number.

### 3.2. Simulation Results

In order to test the proposed implementation, 1024 bit test vectors taken from [20] are employed. The encrypted message and secret keys are passed to the routine for decryption. To determine the performance, the calculation time is evaluated.

With the mentioned algorithms in Section 3.1, it is possible to decrypt $c^{*}$ using LabVIEW correctly. However, it takes a very long time to calculate. The average duration of ten decryption processes is 51.3 s. Since such a long processing time does not allow a proper experiment in a real-life scenario, this method is not suitable for a prototyping platform of a UHF RFID reader.

The LabVIEW built-in resource monitor allows for determining the execution times and numbers of calls of each SubVI. Based on this, the usage in percent of each basic operation called by the decryption blocks (Figure 3) is calculated and listed in Table 2. Since it is called more than $2\times 10^{6}$ times, the add operation consumes the most processing time with a total time of 28.4 s. In order to achieve an acceptable total processing time of a few milliseconds, which means an improvement of several orders of magnitude, only an optimization of the algorithms is not sufficient. For example, the ripple carry adder algorithm would have to be accelerated by a factor of about $10^{4}$. Because this is not possible even with the fastest algorithms, the decryption cannot be implemented in this way in LabVIEW.

## 4. Time-Optimized Implementation Using External Calculations

**Figure 5:**
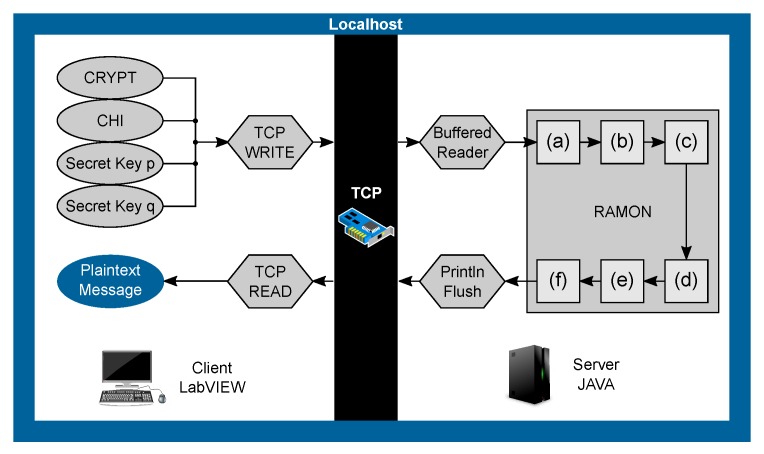
Flow chart of the time-optimized decryption using a TCP/IP connection to interact between LabVIEW and the Java application. CHI, the secret keys *p* and *q* and the crypt $c^{*}$ are transferred from LabVIEW to the Java application via a TCP/IP connection. The decryption process is performed in the Java application. It sends back the plain message *x* to LabVIEW when completed.

Due to the long processing time of the decryption with LabVIEW, a different approach had to be found. However, since the transceiver modules of the PXIe chassis can only be controlled with LabVIEW, and the fully functional protocol of the UHF RFID reader is already available, the use of LabVIEW cannot be avoided. Therefore, the idea is to outsource the decryption to an additional, independent application. The decision for the platform was made in favor of Java, based on its ability to easily handle large numbers with the BigInteger class and previous experience in Java programming. To benefit from the speed advantage of the Java code, the idea is to run a Java application for the calculations in parallel with the LabVIEW program. In order to exchange data between the programs, a TCP/IP connection is employed. After this connection is established, LabVIEW sends the crypt $c^{*}$, the secret keys *p* and *q* as well as CHI and receives only the correct plain message *x* from the Java application. In the proposed implementation, LabVIEW is configured as a Client, while the Java applications provide the TCP/IP server. The flowchart in Figure 5 shows the decryption process executed by both LabVIEW and the Java application, which are communicating via the given TCP/IP connection. The introduction of a TCP/IP connection does lead to a potential point of attack, since the decrypted data is ultimately transmitted unencrypted, even if internally. However, this is the implementation of a prototyping platform, which is used for the development of UHF RFID systems. Thus, the security of this implementation is not in focus, unlike in a commercial RFID reader.

### 4.1. Implementation

#### 4.1.1. LabVIEW

**Figure 6:**
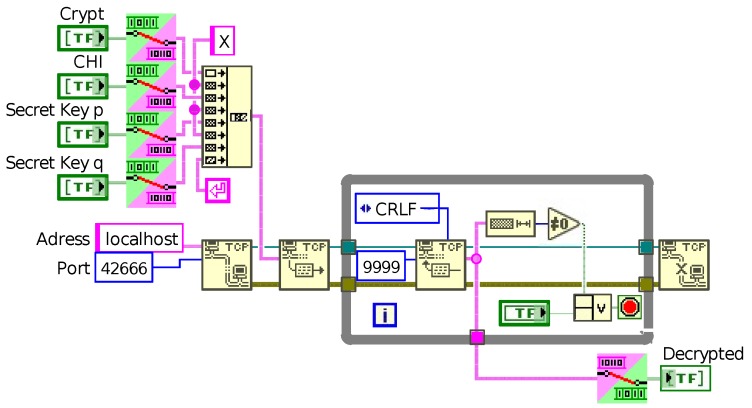
LabVIEW part of the time-optimized decryption process. The crypt, CHI and secret keys are prepared and then transferred via the established TCP/IP connection to the Java application on the localhost. Afterwards, the program waits for a response from the Java application. The plain message is shown at the frontend after reception and the TCP/IP connection is terminated.

Figure 6 shows all necessary steps in LabVIEW to initialize a TCP/IP connection and sends all data to the Java application. Since Java interprets input from a socket as a string, all needed variables like the crypt $c^{*}$, CHI and both secret keys *p* and *q* have to be converted from boolean arrays to strings. This conversion is implemented by simply checking for “true” or “false” in the array and putting together a string of ASCII coded ones and zeros. To send only one message to the Java application for simplicity, all strings are separated by an “X” and merged together, so that Java can later identify all separate variables. The “End-of-Line” added to the end of the message in Figure 6 is required; otherwise, the “Buffered Reader” in Java can not detect the end of the message.

The TCP/IP connection in LabVIEW is easily established by linking the address “localhost” as string (alternatively IP: 127.0.0.1) as well as a port number. With the standard block “TCP write”, the message is then sent to the Java application. To receive a message back, the “TCP read” block is needed. To ensure the receipt of an incoming message, this block is placed inside a loop, which is stopped after a message is detected by checking its length. After receiving the message, LabVIEW closes the connection and finally needs to convert the received string to a boolean array. It is important to mention that this conversion block also deletes all ‘End-of-Line’ symbols attached by Java; otherwise, the result is false. Finally, the boolean array is the correct plain message *x* and can be processed further.

#### 4.1.2. Java

The decryption in the Java application is working like described in Figure 5. First, the Server is initialized with the local host IP address 127.0.0.1 as well as a port number. After successfully opening the server socket, Java enters a “Buffered Read” mode, meaning the application is listening on the port for incoming messages until it receives an “End-of-Line” statement. The Java application then reads the merged message sent by LabVIEW, splits on every “X” and converts the substrings to BigInteger variables. These variables are now given to the RAMON decryption algorithm used from [20], which calculates four, still mixed, possible solutions as BigIntegers.

The “Inverse Mix” function is only described as pseudo code in Ref. [20], so it had to be implemented in Java code. Since this function performs bitwise XOR operations on the individual bytes of the BigInteger, the given BigInteger in base two is converted to an integer array, where each integer represents one byte. Care has to be taken because, in this conversion, the individual bytes of the BigInteger could be interpreted as signed integers, which would lead to an incorrect result. After completing the inverse mix function, CHI is parsed from each of the four possible solutions and compared to the known CHI. Thus, the correct plane message *x* can be selected.

Finally, the correct plain message is sent back to LabVIEW via the TCP/IP connection. Subsequently, the Java application returns to an endless loop while waiting for a new connection to restart the procedure.

### 4.2. Simulation Results

After testing the RAMON Crypto Suite in LabVIEW combined with the Java application using the same test vectors taken from [20], the correct decryption is confirmed. By implementing time measuring commands before and after the computation in the Java application, it is proven that the calculation by the Java server only takes 1.3 ms. The average total execution time of 1000 calculations in LabVIEW combined with the Java application including data transmission via TCP/IP is 2.2 ms. Compared to the implementation in LABView only, the processing time is now in an acceptable range to perform realistic experiments with this prototyping platform.

These findings are similar to the work of [27] where plain LabVIEW implementations of functions for large number arithmetics are compared to C++ implementations loaded into LabVIEW as shared libraries. They find the external C++ libraries to perform calculations significantly faster.

## 5. Conclusions

In order to cope with the rapid developments in the UHF RFID field, easy adaptable readers based on software defined radios are indispensable. This paper shows a way to implement the ISO 29167-19 RAMON decryption on a prototyping reader platform based on National Instruments PXIe chassis. The programming of the PXIe is done with LabVIEW. The first implementation in LABView only has shown that the decryption takes 51.3 s. This would mean that only one tag per minute can be processed. Thus, it is not suitable for a prototyping platform intended to perform realistic experiments in the development of new functionalities in UHF RFID systems. Therefore, a different approach is chosen. LabVIEW still serves as a framework and controls the built-in transceivers. However, it also establishes a TCP/IP connection to a Java application. This Java application performs the decryption. The decrypted plain message is then returned to LabVIEW via a TCP/IP connection and can be further processed. The overall time consumed using LabVIEW combined with the Java application is 2.2 ms. This makes the time-optimized approach 23,318 times faster than the basic implementation in LabVIEW only. The main problem in LabVIEW only is the difficulty of dealing with large numbers as they are commonly used in encryption. Solving this problem by outsourcing the computation to a Java application is not limited to the implementation of the RAMON procedure. The approach can be used for all encryption methods as well as for other procedures handling large numbers in LabVIEW. This way, it is possible to integrate the RAMON encryption into the already existing reader protocol [16] and get a fully functional, easy adaptable reader. It creates completely new possibilities in rapid development especially for UHF RFID systems.

Within IoT, the development of secure batteryless wireless sensor nodes is very interesting. In future studies, RFID transponders that provide encryption will be implemented either in silicon or by using Field Programmable Arrays together with an analog frontend. The presented modular UHF RFID reader then serves as an interface to access such new security features of the tag and test them via the wireless RF link. Additionally, further implementation of security and decryption features by using the same techniques can be performed. Thereby, different approaches for cryptography can be studied and performance in real RFID applications can be investigated.

## Figures and Tables

**Table 1:** Correlation between the LabVIEW SubVI-Blocks and RAMON decryption equations.

SubVI	Equation	Description
(a)	(3)	Calculates real cyphertext *c* from given $c^{*}$, *p*, *q* and *R*
(b)	(3)	Computation of inverse elements $p_{i}$ and $q_{i}$ from secret keys *p* and *q*
(c)	(4)–(5)	Intermediate step to calculate square roots depending on *p* and *q*
(d)	(6)–(9)	Computing term1 and term2 of the root-calculation: $term1=p_{i}\;\cdot \;p\;\cdot \;a_{q}$ $term2=q_{i}\;\cdot \;q\;\cdot \;a_{p}$
(e)	(6)–(9)	Calculating the four roots of the problem
(f)	(-)	Inverse MIX function for demasking to plain messages $x_{1}$,…$x_{4}$

**Table 2:** Breakdown of the calculation duration to the individual blocks from Figure 3. As one can see, the adder consumes the most time due to the high number of calls.

		Number of	Block Usage [%]					
Operation	Time [ms]	executions	(a)	(b)	(c)	(d)	(e)	(f)
Adder	28454	2029269	0.07	26.82	72.96	0.04	0.11	0.00
Shift	6817	1940217	0.06	30.84	68.95	0.09	0.07	0.00
Modulo	3556	1327	0.08	23.06	76.71	0.00	0.15	0.00
Multiply	3588	2420	0.04	37.93	61.86	0.17	0.00	0.00
Subtract	2277	508301	0.08	23.18	76.53	0.00	0.15	0.00
Resize	5413	5088584	5.03	24.99	64.84	0.00	5.11	0.00
Compare	1170	1015246	0.08	23.11	76.67	0.00	0.15	0.00
